# Occurrence, Antibiotic Resistance and Biofilm-Forming Ability of *Listeria monocytogenes* in Chicken Carcasses and Cuts

**DOI:** 10.3390/foods13233822

**Published:** 2024-11-27

**Authors:** Sarah Panera-Martínez, Rosa Capita, Ángela Pedriza-González, María Díez-Moura, Félix Riesco-Peláez, Carlos Alonso-Calleja

**Affiliations:** 1 Department of Food Hygiene and Technology, Veterinary Faculty, University of León, E-24071 León, Spain; 2 Institute of Food Science and Technology, University of León, E-24071 León, Spain; 3 Department of Electrical Engineering and Systems and Automation, School of Industrial, Computer and Aerospace Engineering, University of León, E-24071 León, Spain

**Keywords:** *Listeria monocytogenes*, poultry, antibiotic-resistance, biofilm-forming ability, confocal laser scanning microscopy

## Abstract

A total of 104 samples of chicken meat acquired on the day of slaughter from two slaughterhouses in northwestern Spain were analyzed. These comprised 26 carcasses and 26 cuts from each of the two establishments. An average load of 5.39 ± 0.61 log_10_ cfu/g (total aerobic counts) and 4.90 ± 0.40 log_10_ cfu/g (psychrotrophic microorganisms) were obtained, with differences (*p* < 0.05) between types of samples and between slaughterhouses. Culturing methods involving isolation based on the UNE-EN-ISO 11290-1:2018 norm and identification of isolates by polymerase chain reaction (PCR) to detect the *lmo1030* gene allowed the detection of *Listeria monocytogenes* in 75 samples (72.1% of the total; 50.0% of the carcasses and 94.2% of the cuts). The 75 isolates, one for each positive sample, were tested for resistance against a panel of 15 antibiotics of clinical interest by the disc diffusion method. All isolates belonged to the serogroup IIa (multiplex PCR assay) and showed resistance to between four and ten antibiotics, with an average value of 5.7 ± 2.0 resistances per isolate, this rising to 7.0 ± 2.1 when strains with resistance and reduced susceptibility were taken together. A high prevalence of resistance was observed for antibiotics belonging to the cephalosporin and quinolone families. However, the level of resistance was low for antibiotics commonly used to treat listeriosis (e.g., ampicillin or gentamicin). Nine different resistance patterns were noted. One isolate with each resistance pattern was tested for its ability to form biofilms on polystyrene during 72 h at 12 °C. The total biovolume of the biofilms registered through confocal laser scanning microscopy (CLSM) in the observation field of 16,078.24 μm^2^ ranged between 13,967.7 ± 9065.0 μm^3^ and 33,478.0 ± 23,874.1 μm^3^, and the biovolume of inactivated bacteria between 0.5 ± 0.4 μm^3^ and 179.1 ± 327.6 μm^3^. A direct relationship between the level of resistance to antibiotics and the ability of *L. monocytogenes* strains to form biofilms is suggested.

## 1. Introduction

Poultry constitutes the second most widely produced and eaten type of meat in Europe, its consumption being especially notable in some countries, such as England, Ireland, Portugal, Spain and France [1]. It is expected that in 2024, there will be a marked increase in the production and consumption of fresh poultry meat worldwide, representing 3.5% more than in 2023 and 4.4% more than in 2022 [2]. In recent years, great efforts have been made to reduce the incidence of diseases transmitted by meat and meat products, such as the implementation of Hazard Analysis and Critical Control Points (HACCP) principles [3]. Despite this, the intrinsic characteristics of fresh meat, in particular its abundant nutrient content and strong water activity, make it a food with high levels of microbial contamination [4,5]. Poultry meat continues to be a major reservoir of both spoilage microorganisms [6,7] and pathogens, such as *Listeria monocytogenes* [8,9].

The *Listeria* genus is made up of Gram-positive bacteria, facultative anaerobes with the shape of a coccobacillus, which do not have a capsule or form spores [10]. Thirty species have been described [11], although *L. monocytogenes* is the one responsible for the vast majority of cases of human and animal listeriosis [12]. Meat and meat products play an important role in the transmission of this disease, owing to the possible presence of the microorganism in animals and the contamination that occurs during slaughtering and processing, when there is contact between meat and the various surfaces and pieces of equipment in industrial plants [13].

The pathogenicity of some bacterial species, for example, *L. monocytogenes*, is directly related to their virulence but also to other mechanisms that allow bacteria to persist and grow in stressful environments, such as their ability to form biofilms and their level of resistance to antibiotics [14,15]. Some strains of *L. monocytogenes* are capable of forming robust biofilms on different surfaces in food processing plants [16], persisting in these environments because of the strong tolerance sessile cells have to the disinfectants used during sanitization of facilities [17]. Furthermore, the use of sub-lethal concentrations of disinfectants can be counterproductive since low doses of biocides are linked to an increase in tolerance to these substances and resistance to antibiotics, in addition to a greater bacterial capacity to form biofilm [12]. Also, the misuse or excessive use of antibiotics in numerous areas, including medical, veterinary, and agricultural environments, has caused selective pressure that has contributed to the emergence and spread of antibiotic-resistant strains of *L. monocytogenes* [18].

In light of this, the present research work was undertaken with the objectives of (1) to know the hygienic and sanitary condition of chicken meat obtained on the day of slaughter from two poultry abattoirs in north-western Spain; (2) to discover the prevalence of *L. monocytogenes* in this meat; (3) to determine the antibiotic resistance in *L. monocytogenes* isolates; and (4) to found a relationship between the antibiotic resistance patterns found in *L. monocytogenes* isolates with their ability to form biofilms.

## 2. Methods and Procedures

### 2.1. Sampling and Determination of the Microbiological Quality of Chicken Meat

A total of 104 samples, comprising 52 carcasses and 52 chicken cuts, were obtained on the day of slaughter from two different abattoirs located in north-western Spain, A and B, with 26 carcasses and 26 cuts taken from each establishment. Samples were collected between March 2022 and February 2023. The samples were analyzed to determine their microbiological quality by taking 25 g of skin, which were homogenized in 225 mL of 0.1% peptone water (CM0009, Oxoid, Ltd., Hampshire, UK) for two minutes (Masticator Silver Panoramic, IUL Instruments, Barcelona, Spain). Total aerobic counts (TAC) and psychrotrophic microorganisms were determined for each sample by culture-dependent methods. To do this, decimal dilutions of each homogenate were made in 0.1% peptone water, and 100 µL of three non-consecutive dilutions were plated in duplicate by surface spread on plate count agar (PCA, CM03225, Oxoid, Ltd., Hampshire, UK). The plates were incubated at 30 °C for 72 h to determine TAC levels [19] and at 7 °C for 10 days to count psychrotrophic microorganisms [20].

### 2.2. Prevalence of Listeria monocytogenes

The methodology described in the UNE-EN-ISO 11290-1:2018 norm [21] was used for the detection of *L. monocytogenes*. From each sample, 25 g of skin were homogenized for two minutes with 225 mL of Half-Fraser broth (CM0895, Oxoid). After incubation at 30 °C for 24 h, 100 µL of the homogenate were transferred to tubes with 10 mL of Fraser broth (CM0895, Oxoid), which were incubated at 37 °C for a further 24 h. Thereafter, a quantity of 20 µL of the broth was plated on Petri dishes with OCLA medium (CM1084, Oxoid Chromogenic *Listeria* Agar), these being incubated for 48 h at 37 °C.

From each positive sample, three colonies with the morphology and characteristics typical of *L. monocytogenes* were taken (blue-green colonies with an opaque halo); these then being incubated at 37 °C for 24 h in tubes with 9 mL of TSB (CM0129B, Tryptone Soya Broth, Oxoid). To preserve these isolates, 1.2 mL of the culture were transferred to sterile vials with 300 µL of glycerol (151339.1211, PanReac AppliChem, Barcelona, Spain) and kept at −30 °C until further use.

These frozen strains were streaked onto TSA plates (CM0131B, Tryptone Soy Agar, Oxoid) and incubated at 37 °C for 24 h, after which DNA extraction was carried out by collecting between three and five colonies with a sterile wire loop. These colonies were placed in an Eppendorf tube with 50 µL of tris-ethylenediamine-tetra-acetic acid (TE) buffer prepared as follows: 10 mM Tris-HCl (A2264,1000, PanReac AppliChem) with 1 mM EDTA (34549, Fluka, Steinheim, Germany) and adjusting the pH at 8.0. The tubes were kept at 99 °C for 15 min, then 200 µL of Milli-Q water was added to each tube, followed by centrifuging for 5 min at 13,000 rpm and 4 °C. The supernatant, with the DNA in suspension, was stored at −20 °C until used.

### 2.3. Identification and Serogrouping of Isolates

Confirmation of the isolates, comprising three colonies from each positive sample, was performed by conventional PCR by detecting a 509 bp fragment of the *L. monocytogenes lmo1030* gene using the following pair of primers: Forward Primer 3′-GCTTGTATTCACTTGGATTTGTCTGG-5′ and Reverse Primer 3′-ACCATCCGCATATCTCAGCCAACT-5′ [22]. The reaction was carried out in a total volume of 25 µL, of which 5 µL corresponded to the extracted DNA and the rest to the master mix: 2.5 µL of NH_4_ buffer (10×, 102030, BIORON GmbH, Ludwigshafen, Germany), 1.5 µL MgCl_2_ (100 mM, 102030, BIORON), 0.5 µL each of deoxyribonucleotide triphosphate (10 mM, E0503-02, EURx Sp. z o.o., Gdańsk, Poland), 0.5 µL of each primer (25 µM, Isogen Life Science, Barcelona, Spain), 1.25 U of polymerase enzyme (101005, BIORON) and Milli-Q water to complete a total volume of 20 µL [9,23].

The amplification of the genetic material was carried out in a ProFlex^TM^ thermal cycler (Applied Biosystems, Waltham, MA, USA), programmed as follows: five minutes at 94 °C, 35 amplification cycles and five minutes at 72 °C. Each cycle was composed of three steps: denaturation of the DNA at 94 °C for 30 s, annealing of the primers at 62 °C for 30 s, and elongation of both DNA strands at 72 °C for 45 s. Visualization of the results was performed by electrophoresis in 1.0% agarose gel (604005, BIORON) in 1× tris-acetate-EDTA buffer, stained with 1× SimplySafe dye (E4600-01, EURx). Development was carried out in a Gel Doc EZ System transilluminator (Bio-Rad, Hercules, CA, USA). A molecular weight marker (Perfect Plus 1 kb DNA Ladder, E3131-02, EURx), a negative control (sample without DNA) and a positive control (*L. monocytogenes* DNA) were included in all gels.

*L. monocytogenes* strains (one isolate from each positive sample) were further classified in PCR serogroups with a multiplex PCR assay in accordance with the method previously described [24].

### 2.4. Phenotypic Characterization of Antibiotic Resistance

The resistance of the isolates is identified as *L. monocytogenes* and classified in PCR serogroups were studied using the disc diffusion technique as described by the Clinical and Laboratory Standards Institute [25] with a panel of 15 antibiotics of clinical importance (Table 1). Using the frozen strains, tubes containing 9 mL of MHB (CM0405B, Mueller Hinton Broth, Oxoid) were inoculated and then incubated for a period of between 6 h and 8 h. When the cultures were in the exponential growth phase, antibiograms were performed on plates with MHA medium (CM0337B, Mueller Hinton Agar, Oxoid). This was achieved by inoculating MHA plates with a sterile cotton swab previously soaked in the culture; five antibiotic discs were then placed on each plate. The plates were incubated at 37 °C for 24 h, after which the inhibition zones were measured, allowing the classification of the strains as susceptible, of reduced susceptibility (intermediate) or resistant, according to the standards set by the European Committee on Antimicrobial Susceptibility Testing [26] and Clinical and Laboratory Standards Institute [25,27]. Detailed information about the standards and controls used as reference is provided in Table 1.

### 2.5. Biofilm-Forming Capacity

One *L. monocytogenes* isolate of each antibiotic resistance phenotype found was tested for its ability to form biofilms in order to determine whether the two variables (antibiotic resistance and biofilm-forming ability) were related. The biofilms were produced using the method previously described by Capita et al. [28], with some modifications. This involved incubation of the *L. monocytogenes* isolates in TSB at 37 °C for 24 h, with two decimal dilutions being made in the same culture broth so that an approximate final concentration of 10^6^ cfu/mL was obtained. A volume of 250 μL of this culture was deposited in wells of microtiter plates with polystyrene optical bottom (165305, 96 Well Black/Clear Bottom Plate, Thermo Fisher Scientific, Newington, NH, USA). These plates were incubated at 12.0 °C ± 0.5 °C for 60 min, and the wells were subsequently washed with a 150 mM NaCl solution (31434-500G-R, Sigma-Aldrich Co., St. Louis, MO, USA) with the aim of eliminating non-adherent bacteria. Subsequently, 250 μL of sterile TSB was added, and the plates were incubated at 12.0 °C ± 0.5 °C for 72 h. After incubation, two consecutive washes were performed with 250 μL of 150 mM NaCl, leaving the biofilms that had formed at the bottom of the plate. The wells were filled with 250 μL of a solution composed of TSB and the fluorescent dyes SYTO-9, which stains both live and dead cells, and propidium iodide (PI), which stains only dead cells, at a 1× concentration (1:1000). Both stains came from the BacLight Viability Kit (L7012, Invitrogen, Carlsbad, CA, USA). Microtiter plates were incubated in the dark at 37.0 °C ± 0.5 °C for 20 min to promote penetration of fluorescent components into the bacterial cells.

Images of biofilms were obtained with a Zeiss LSM 800 Airyscan confocal laser scanning microscope (Carl Zeiss, Jena, Germany) and ZEN 2.6 software. All biofilms were scanned at 400 Hz and observed with a 63× (0.8 NA) oil-immersion objective lens. Fluorescence was detected by excitation at 488 nm, and emissions were collected at 590/50 for SYTO9 and 650LP for PI. Three independent experiments were carried out on different days, with each strain using different microtiter plates to study the structure of biofilms. For each of the three wells occupied by any given strain, three stacks of horizontal plane images (512 × 512 pixels, corresponding to 126.8 × 126.8 μm) were acquired from three different randomly chosen areas in the well with a z step of 1.0 μm. Consequently, a total of 81 CLSM images were obtained (9 isolates × 3 replicates × 3 zones from each well). The original Zeiss files (CZI format) were imported into the IMARIS 9.1 software package (Bitplane, Zurich, Switzerland) to analyze the obtained images. Biovolume values (µm^3^) for biofilms of each isolate, replicate and zone tested were calculated within IMARIS 9.1, obtaining a mean value for each antibiotic resistance pattern studied.

### 2.6. Statistical Analysis

The bacterial count data, together with those relating to the biovolume of the biofilms, were studied by employing analysis of variance (ANOVA) techniques, with separation of means through the use of Duncan’s multiple range test. The data concerning the prevalence and antibiotic resistance of *L. monocytogenes* were analyzed using exact Chi-square tests. The correlation between the bacterial amount (TAC and psychrotrophic microorganisms) and the occurrence of *L. monocytogenes* was tested using Pearson’s correlation. All tests were carried out with the RStudio software package [29], with significant differences set at a probability level of 95% (*p* < 0.05).

## 3. Results

### 3.1. Microbiological Quality of Chicken Meat

The mean values, expressed as log_10_ cfu/g, which were recorded were 5.39 ± 0.61 for total aerobic counts (TAC) and 4.90 ± 0.40 for psychrotrophic microorganisms (*p* < 0.05). In both microbial groups, significant differences were observed that were dependent upon the type of sample, whether carcasses or cuts and the slaughterhouse, whether abattoir A or abattoir B. When the counts from both slaughterhouses were taken together, TAC and psychrotrophic load were higher for the cuts than for the carcasses. When the four groups of samples (two types of samples × two slaughterhouses) were considered separately, the highest TAC values were obtained for cuts from abattoirs A and B, and the lowest values were found in carcasses from abattoir B. With respect to psychrotrophic microorganisms, the highest degree of contamination was observed in cuts from abattoir B, even though carcasses from this same slaughterhouse showed the lowest counts (Table 2).

### 3.2. Occurrence of Listeria monocytogenes

**Figure 1:**
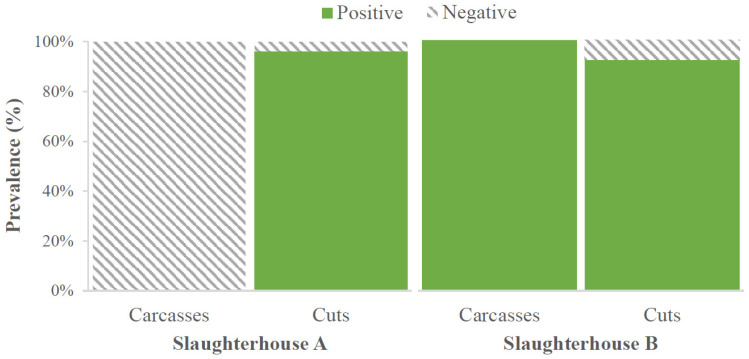
Prevalence of *Listeria monocytogenes* by type of sample (carcasses or cuts) and slaughterhouse (A or B).

Colonies with the typical appearance and morphology of *L. monocytogenes* were isolated from 75 of the 104 samples analyzed (72.1%): 26 carcasses (representing 50.0% of that type of sample) and 49 cuts (94.2%). All the colonies isolated (225 in total) were confirmed as *L. monocytogenes* through detection of the *lmo1030* gene by PCR. The data for pathogen prevalence differed considerably (*p* < 0.05) by type of sample and slaughterhouse concerned (Figure 1). It was striking that none of the carcasses from abattoir A were contaminated with *L. monocytogenes*, while in the other three groups of samples, comprising cuts from abattoir A and carcasses and cuts from abattoir B, more than 90% of the samples were positive.

Samples were grouped by abattoirs to analyze the correlation between the bacterial amount (total aerobic counts and psychotropic microorganisms) and the occurrence of *L. monocytogenes*. The mean values were higher for TAC in samples of abattoir A that were positive to *L. monocytogenes*, with an average of 5.87 ± 0.62 log_10_ cfu/g, while the lowest values (5.13 ± 0.32 log_10_ cfu/g) were recorded for samples without *L. monocytogenes* of the same abattoir. No significant differences were observed for psychrotrophic microorganisms (*p* > 0.05). The correlation tested between the bacterial amount (0.255 for TAC and 0.182 for psychrotrophic microorganisms) and the occurrence of *L. monocytogenes* did not show significant (*p* > 0.05) results for any of the bacterial groups studied.

### 3.3. Serogruping and Phenotypic Characterization of Antibiotic Resistance

All *L. monocytogenes* isolates tested (75) belonged to serogroup IIa and showed resistance to between 4 and 10 of the 15 antibiotics tested, with 9 different resistance phenotypes being recorded (Table 3). None of the isolates was pan-susceptible or resistant to less than 4 antibiotics. Most resistance patterns were present in only one of the isolates. However, it is worth highlighting the existence of two main phenotypes: OX-FOX-CTX-FEP, shown by 37 of the 75 isolates (48.7% of the total) and OX-FOX-CTX-FEP-CN-E-RD-TE, noted in 16 isolates (21.1%). An average value of 5.7 ± 2.0 resistances per isolate was registered, although this value rose to 7.0 ± 2.1 when resistant strains and those with reduced susceptibility were taken together. Of the 1125 tests undertaken involving the 75 isolates identified as *L. monocytogenes* and the 15 antibiotics evaluated, 425 (37.8%) demonstrated resistance, 100 (8.9%) displayed reduced susceptibility, and 600 (53.3%) showed susceptibility. All strains showed resistance to between two and eight different antibiotic categories, including aminoglycosides, rifamycins, penicillins, cephalosporines, macrolides, fluoroquinolones, sulfonamides and tetracyclines.

**Figure 2:**
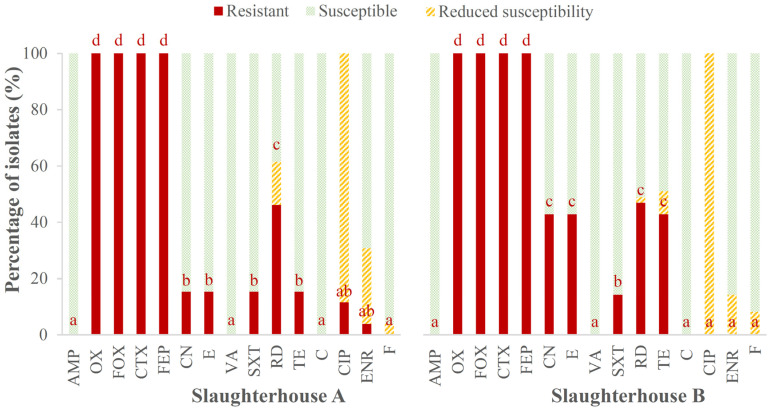
Percentage of *Listeria monocytogenes* isolates with resistance, reduced susceptibility or susceptibility to each antibiotic tested. Antibiotics (the data from the two slaughterhouses are compared separately) that do not share any letters present significant differences one from another (*p* < 0.05). AMP (ampicillin; 10 µg), OX (oxacillin; 1 µg), FOX (cefoxitin; 30 µg), CTX (cefotaxime; 30 µg), FEP (cefepime; 30 µg), CN (gentamycin; 10 µg), E (erythromycin; 15 µg), VA (vancomycin; 30 µg), SXT (trimethoprim-sulfamethoxazole; 25 µg), RD (rifampicin; 5 µg), TE (tetracycline; 30 µg), C (chloramphenicol; 30 µg), CIP (ciprofloxacin; 5 µg), ENR (enrofloxacin; 5 µg), F (nitrofurantoin; 300 µg).

Regardless of their origin (type of sample or abattoir), the majority of the isolates were resistant to cephalosporines and some penicillins but susceptible to other penicillins, glycopeptides and phenicols. With data from both slaughterhouses taken together, it can be stated that all isolates were resistant to OX, FOX, CTX and FEP and susceptible to AMP, VA and C. Furthermore, approximately 15.0% of the isolates from both slaughterhouses were resistant to SXT, between 46.2% and 46.9% to RD and between 3.9% and 8.2% had reduced susceptibility to F. The percentages of isolates resistant to the rest of the antibiotics showed differences between the two slaughterhouses (*p* < 0.05). Of the isolates from abattoir A, 15.4% were resistant to CN, E and TE, while this value rose to 42.9% in the case of abattoir B (Figure 2).

All of the isolates from abattoir B had reduced susceptibility to CIP, while in abattoir A, 11.5% of the isolates were resistant, and the remainder (88.5%) had reduced susceptibility to this antibiotic. Finally, a very small percentage of the isolates from abattoir A (3.8%) were resistant to ENR, but this figure rose to 30.8% of the isolates if resistance and reduced susceptibility were taken together, while in abattoir B, only 14.3% of the isolates had reduced susceptibility to this antibiotic, and no isolate showed resistance.

### 3.4. Biofilm Formation

**Figure 3:**
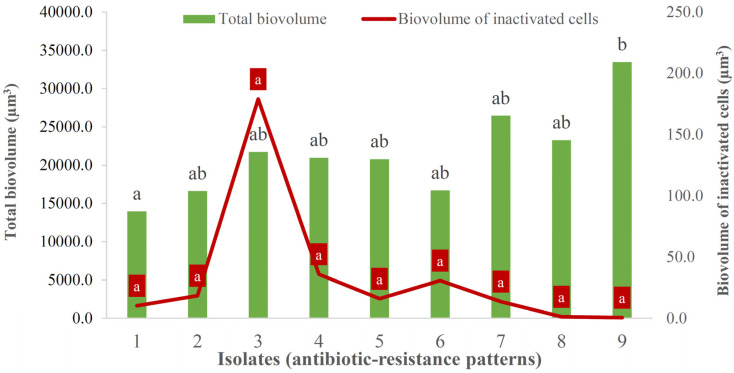
Total biovolume (green bars; left-side *y*-axis) and biovolume of inactivated bacteria (red line; right-side *y*-axis) of the biofilms formed on polystyrene (72 h at 12 °C) for each *Listeria monocytogenes* isolate tested. Data (total biovolume and biovolume of inactivated bacteria were compared separately) with no letters in common are significantly different (*p* < 0.05). One isolate from each of the resistance phenotypes was studied: (1) OX-FOX-CTX-FEP; (2) OX-FOX-CTX-FEP-RD; (3) OX-FOX-CTX-FEP-CIP; (4) OX-FOX-CTX-FEP-SXT; (5) OX-FOX-CTX-FEP-SXT-RD; (6) OX-FOX-CTX-FEP-CIP-ENR; (7) OX-FOX-CTX-FEP-CN-E-RD-TE; (8) OX-FOX-CTX-FEP-CN-E-SXT-RD-TE; (9) OX-FOX-CTX-FEP-CN-E-SXT-RD-TE-CIP. OX (oxacillin; 1 µg), FOX (cefoxitin; 30 µg), CTX (cefotaxime; 30 µg), FEP (cefepime; 30 µg), CN (gentamycin; 10 µg), E (erythromycin; 15 µg), SXT (trimethoprim-sulfamethoxazole; 25 µg), RD (rifampicin; 5 µg), TE (tetracycline; 30 µg), CIP (ciprofloxacin; 5 µg), ENR (enrofloxacin; 5 µg).

All *L. monocytogenes* isolates tested, a total of 9, one from each resistance phenotype, were capable of forming biofilms on polystyrene under the experimental conditions set, 72 h at 12 °C. The total biovolume recorded in the observation field of 16,078.24 μm^2^ ranged between 13,967.7 μm^3^ ± 9065.0 μm^3^ and 33,478.0 μm^3^ ± 23,874.1 μm^3^, with the biovolume of inactivated bacteria lying between 0.5 μm^3^ ± 0.4 μm^3^ and 179.1 μm^3^ ± 327.6 μm^3^, as shown in Figure 3. In terms of total biovolume, only significant differences (*p* < 0.05) were recorded between the first pattern (resistance to four antibiotics: OX-FOX-CTX-FEP) and the last (resistance to ten antibiotics: OX-FOX-CTX-FEP-CN-E-SXT-RD-TE-CIP).

**Figure 4:**
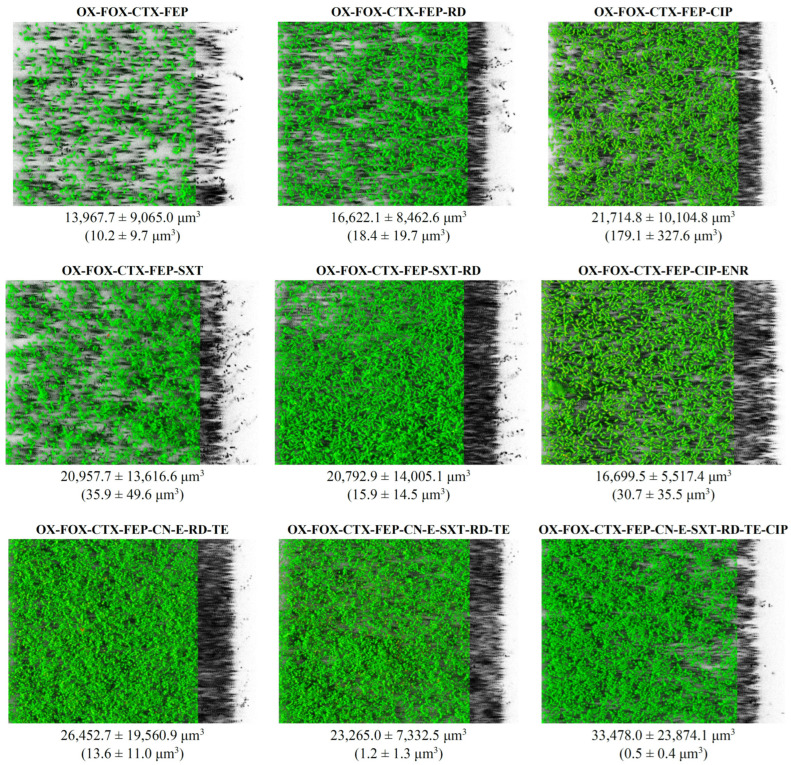
Three-dimensional reconstructions of the biofilms formed on polystyrene (72 h; 12 °C) by *Listeria monocytogenes* isolates of different antibiotic resistance patterns. The total biovolume (μm^3^) observed by SYTO9 green staining is not in parentheses, while the biovolume (μm^3^) of inactivated bacteria observed after PI red staining is shown in parentheses. The images (126.8 μm × 126.8 μm) were reconstructed with the IMARIS 9.1 program, with virtual projections of the shadow on the right. OX (oxacillin; 1 µg), FOX (cefoxitin; 30 µg), CTX (cefotaxime; 30 µg), FEP (cefepime; 30 µg), CN (gentamycin; 10 µg), E (erythromycin; 15 µg), SXT (trimethoprim-sulfamethoxazole; 25 µg), RD (rifampicin; 5 µg), TE (tetracycline; 30 µg), CIP (ciprofloxacin; 5 µg), ENR (enrofloxacin; 5 µg).

The three-dimensional reconstructions of the nine biofilms are shown in Figure 4, in which it may be seen that all the isolates were capable of producing rough biofilms that covered a large part of the polystyrene surface. The results obtained reveal a marked variability in the structure and architecture of the biofilms, with more compact structures being observed in the final three images, corresponding to patterns of resistance to a greater number of antibiotics, while biofilms were less dense in the earlier images, corresponding to patterns of resistance to a fewer number of antibiotics.

## 4. Discussion

### 4.1. Microbiological Quality of Chicken Meat

TAC and psychrotrophic microorganisms are frequently used to estimate the microbiological quality of foods [30]. Although the levels of these two microbial groups are not directly related to the risk to human health, their quantification allows the determination of whether good hygiene practices have been implemented during the production of food [31].

The average value for TAC recorded in the present study (5.39 ± 0.61 log_10_ cfu/g) is similar to those previously found by other authors in poultry meat and meat derivatives, the figures (log_10_ cfu/g) being: 5.10 ± 0.59 in poultry legs [30], between 5.79 and 5.85 in chicken cuts (legs, wings and giblets) and processed chicken products (hamburgers and sausages) [6], 5.04 ± 0.51 in chicken burgers [32], 5.02 ± 0.50 in chicken meat products [33], 6.91 ± 2.13 in fresh chicken meat samples [34] and 5.46 ± 1.10 in fresh chicken carcasses [35]. However, the counts registered in the research being reported here were lower than those previously found in studies also carried out in the northwest of the Iberian Peninsula in minced chicken meat, with values of 7.53 ± 1.02 log_10_ cfu/g in [24].

On the other hand, the study of psychrotrophic microorganisms is crucial in the case of raw foods preserved by refrigeration, such as poultry products [36,37]. In the present study, mean counts of 4.90 ± 0.40 log_10_ cfu/g of psychrotrophic microorganisms were found, while in other similar works carried out on poultry meat or meat derivatives, the following values were obtained (log_10_ cfu/g): 4.34 ± 0.77 in poultry legs [31], between 5.96 and 7.87 in chicken cuts (legs, wings and giblets) and processed chicken products (hamburgers and sausages) [6], between 3.8 and 5.8 in poultry retail meat [30] and 2.87 ± 0.60 in chicken meat products [33]. As in the case of TAC, the levels of psychrotrophic microorganisms seen in the present study were lower than those previously noted in minced chicken meat (7.13 ± 1.07 log_10_ cfu/g) [24].

The differences observed between the values for TAC and for psychrotrophic microorganisms (lower in the latter microbial group) may be due to the fact that the samples for analysis were taken on the same day the animals were slaughtered. It is possible that if testing had been carried out after several days of refrigerated storage, higher levels of psychrotrophic microorganisms than of TAC might have been recorded. On the other hand, the differences between carcasses and cuts (the latter presented a higher degree of contamination) may be due to the handling processes required during cutting, which are likely to favor contamination with microorganisms [38,39]. Furthermore, for both TAC and psychrotrophic microorganisms, levels ranged between 3.92 and 7.38 log_10_ cfu/g. Only three samples presented counts higher than 7 log_10_ cfu/g for TAC (2.9% of the total) and just two (1.9%) in the case of psychrotrophic microorganisms. In no instance was a value of 8 log_10_ cfu/g exceeded. On the basis of the reports consulted [32], most of the samples were of satisfactory quality (counts of TAC and psychrotrophic microorganisms between 4 and 7 log_10_ cfu/g), while the rest of the samples were unacceptable in quality (TAC values higher than 7 log_10_ cfu/g) [40].

### 4.2. Prevalence and Serogroups of Listeria monocytogenes

In the present study, *L. monocytogenes* was confirmed in 72.1% of the samples analyzed, a value higher than that observed by other authors in similar studies of carcasses, cuts or poultry meat preparations. In these, the prevalences of cuts reported were: 12.7% [41], 17.9% [42], 18.0% [43], 18.2% [44], 19.2% [45], 34.3% [46], 48.7% [47], 50.0% [23], 60.0% [48], and 70.0% [49]. Those for whole carcasses were: 3.61% [50], 8.33% [51], 15.8% [52], 18.7% [53], 19.3% [54], 21.0% [55], 24.5% [56], 32.0% [8], 40.0% [57], 41.0% [58], and 56.8% [44]. Finally, in products made from poultry meat, the levels reported were 9.0% [59], 16.0% [60], 23.8% [61], 33.3% [62], 56.0% [9], and 70.0% [35]. The differences observed between studies could be due to the geographical area where the analyses were carried out, the different origins of the animals, or the presence of persistent strains in the slaughterhouses, among other causes.

The prevalence of *L. monocytogenes* noted in the literature consulted in relation to other types of meat was also considerably lower than the values registered in the present study. In pork and pork preparations, the figures reported were 3.2% [53], 6.0% to 10.0% [63], 10.3% [64], 13.7% [50], and 20.6% [65]. In beef and beef preparations, the prevalences noted were 10.3% [64], 12.2% [65], 13.2% [53], 16.7% [51], and 33.3% [66]. Finally, a figure of 6.1% [50] was quoted for sheep meat. The differences observed in the prevalence of *L. monocytogenes* in different types of meat, generally higher in the case of poultry, may be due to fecal contamination during evisceration since birds are relatively frequently asymptomatic carriers of this microorganism [54,56].

It is striking that there were considerable differences in the prevalence values observed between the two slaughterhouses sampled, with 48.1% of positive samples in abattoir A and 96.2% in abattoir B, and between the types of samples analyzed, with 50.0% positive samples in whole carcasses, but 94.2% in cuts. These differences may be due to the origin of the animals themselves since each slaughterhouse obtained them from different sources, or to cross-contamination phenomena when there was contact in the slaughterhouse with equipment or staff as a consequence of bad practice during slaughtering, cutting, or both [7,42].

Most of the results listed above have been obtained from fresh poultry meat or poultry products that will have a cooking process prior to consumption, which could mitigate the hazard. It is important to highlight that the data from this study shows the level of contamination with *L. monocytogenes* on the day of slaughter (without any days of storage), so it reflects the microbial status of the samples right before entering the distribution chain. The impact on public health depends on the step within which food has been contaminated with the pathogen. For example, if contaminated food enters a retail establishment, the possibility that other foods become cross-contaminated significantly increases [67]. Also, the presence of *L. monocytogenes* at the initial steps of the poultry meat production chain is of particular concern since its ability to grow at temperatures close to 0 °C can increase its concentration over the course of storage [9].

All strains were assigned to the serogroup IIa. In a previous research work [24], this serogroup was the most prevalent in minced chicken meat from both Spain and Portugal (84.4% and 56.0% of isolates, respectively). The strains in this serogroup (which includes serovars 1/2a and 3a) have shown extensive distribution in food-stuffs and food-processing environments around the world, thereby indicating its high ecological adaptability [24].

### 4.3. Phenotypic Characterization of Antibiotic Resistance

Considering the whole set of strains and antibiotics tested, it may be stated that 37.8% resistance was observed. There was an average of 5.7 ± 2.0 resistances per strain, which would rise to 7.0 ± 2.1 if resistance and reduced susceptibility were lumped together. These figures are similar to those previously observed in *L. monocytogenes* strains isolated from poultry meat preparations, where there was 38.9% resistance and an average of 5.8 ± 1.6 resistances per strain [9]. This was also true for poultry cuts, with 34.1% resistance and an average of 5.1 ± 1.3 resistances per strain [23], and for minced chicken meat, with 37.3% resistance and an average of 5.7 ± 1.2 resistances per strain [24]. However, the level of resistance observed in the present study is higher than what was noted by Parra Flores et al. [68], who recorded 2.7% resistance in *L. monocytogenes* strains isolated from ready-to-eat products.

In the present study, all isolates presented resistance to between 4 and 10 antibiotics. These figures are similar to those previously observed in meat preparations, with resistance to between 5 and 15 antibiotics [9], cuts of poultry, in which the figures were between four and nine [23], and minced chicken meat, at between three and eight antibiotics [24]. The multidrug-resistant phenotype (MDR) is defined as acquired non-susceptibility to at least one agent in three or more antimicrobial categories, with one or more antibiotics from each category being applied [69]. In the present study, 34.7% of the isolates tested showed resistance to between 3 and 7 different antibiotic categories: aminoglycosides, rifamycins, penicillins, macrolides, fluoroquinolones, sulphonamides and tetracyclines. It should be noted that most strains of *L. monocytogenes* show intrinsic resistance to cephalosporines [70], so this group of antibiotics was not taken into account to determine the percentage of isolates having MDR phenotype.

Other authors have found lower percentages of multi-resistant *L. monocytogenes* strains: 2.9% of strains isolated from meat products and processing environments [71], 14.0% of those from fish [72], 18.6% of strains found in poultry meat [56], and 25.0% of strains isolated from chicken carcasses [57]. In other contexts, figures were higher, with 46.7% of strains isolated from ready-to-eat products [73], 60.2% of strains found in poultry meat products [62], 85.7% of those discovered in milk and dairy derivatives [74], and 84.0% of strains isolated from poultry meat [56] showing multiple resistances. The presence of multidrug-resistant *L. monocytogenes* in food of animal origin poses a high risk of infection for consumers due to its difficulty to treat in case of invasive listeriosis [75].

Of the nine resistance phenotypes found, four were particularly representative, being observed in 49.9% (OX-FOX-CTX-FEP), 21.3% (OX-FOX-CTX-FEP-CN-E-RD-TE), 12.0% (OX-FOX-CTX-FEP-RD) and 10.7% (OX-FOX-CTX-FEP-CN-E-SXT-RD-TE) of the isolates. The antibiotics concerned have received classifications of “critically important” (CN, RD, CTX, FEP, E) or “highly important” (OX, FOX, SXT, TE) in human medicine [76] and of “critical importance” (CN, OX, E, TE) or “high importance” (RD) in veterinary medicine [77]. These results are consistent with those previously obtained in poultry meat preparations, cuts of poultry and minced chicken meat, where more than 90.0% of the isolates presented resistance to OX, FOX, CTX and FEP [9,23,24]. Other authors also found *L. monocytogenes* strains resistant to some of these antibiotics in ready-to-eat products. Thus, Şanlıbaba et al. [78] isolated strains with resistance to OX (94.1%), RD (47.1%), SXT (5.9%) and TE (17.7%), while Arslan and Özdemir [73] found strains with resistance to SXT (13.3%), TE (6.7%) and RD (6.7%). For their part, Capita et al. [70] described strains of *L. monocytogenes* isolated from red meat and poultry with a high prevalence of resistance to six antibiotics: FOX (77.8%), CTX (62.5%), FEP (73.6%), NA (97.2%), F (51.4%) and OX (93.1%).

Despite the considerable resistance to antibiotics of *L. monocytogenes* recorded in the present study, it should be noted that the greatest prevalence of resistance was to antibiotics from the cephalosporin and quinolone families, which generally have sparse effects against this bacterium [79], and so are not usually used in clinical practice to treat listeriosis. On the other hand, the resistance values registered in this study relative to ampicillin (0.0%) and gentamycin (15.4% and 42.9% in slaughterhouses A and B, respectively), these being antibiotics commonly used to treat infections caused by this pathogen [12,80], were not excessively high. These data are similar to those reported by other authors, who also point out the low prevalence (less than 25.0%) of strains with resistance to these antibiotics [68,71,72,73,74,78]. It is also worth noting that in abattoir B, higher prevalences of strains with resistance to CN, E and TE were found than in abattoir A. These differences do no more than reflect the obvious relationship that exists between different antimicrobial resistance profiles and the environment in which the microorganisms displaying them are isolated.

### 4.4. Biofilm Formation

The ability of nine *L. monocytogenes* isolates, one for each resistance phenotype, to form biofilm on polystyrene surfaces was determined after incubation in TSB at 12 °C for 72 h. This material is frequently used in the facilities and equipment of the food industries [81]. In all cases, the biofilms were made up of several layers of cells. The isolate of the OX-FOX-CTX-FEP phenotype had the lowest biovolume, at 13,967.7 ± 9065.0 µm^3^. In contrast, the isolate with the highest total biovolume corresponded to the OX-FOX-CTX-FEP-CN-E-SXT-RD-TE-CIP phenotype, with a mean value of 33,478.0 ± 23,874.1 µm^3^. This fact is striking since one isolate was from the phenotype with the lowest number of resistances, four, and the other had the highest, ten.

It has been demonstrated that sessile bacteria are more resistant than planktonic cells to treatment with biocides and antibiotics because of the structural characteristics of the biofilm itself, making their elimination difficult [82,83,84]. In the present study, the antibiotic resistance of each isolate was determined prior to the formation of biofilms, so the results may indicate the existence of a prior relationship between the levels of antibiotic resistance and the capacity for biofilm formation, such that the more resistant the bacteria is, the greater its ability to form biofilm. Such a finding coincides in part with those previously observed with *L. monocytogenes* strains isolated from joints of poultry, in which a limited correlation (r = 0.227; *p* > 0.05) was observed between the percentage of resistance per isolate and the biofilm-forming ability [23]. A similar linkage has also been verified in other bacterial species, such as *Enterococcus* spp. [85] or *Escherichia coli* [86]. However, other studies carried out with strains of *Acinetobacter baumannii* [87] or *Salmonella enterica* [88] showed that strains with a higher level of resistance to antibiotics formed weaker biofilms. The discrepancies between studies suggest the need to carry out new studies to clarify the relationship between antibiotic resistance and the ability to form biofilms.

The main challenge or limitation of this research work has been finding suitable antibiotic resistance criteria for *L. monocytogenes* since the existing standards only include some antibiotics. As this is a study to determine the relationship between the degree of antibiotic resistance and the ability to form biofilms, we believe that it would be a mistake to take into account only the small number of antibiotics that are included in the standards. Therefore, in some cases, existing reference values for other Gram-positive microorganisms have been used. On the other hand, as our objective was to obtain as much information as possible on the level of resistance of the strains, antibiotics to which *L. monocytogenes* is intrinsically resistant (such as cephalosporins) were also studied to determine if the strains were resistant or only had reduced susceptibility to such compounds.

Another limiting factor of this study was that all the strains analyzed belonged to the same *L. monocytogenes* serogroup (IIa). However, this is also a positive aspect since a relationship can be established between the resistance to antibiotics and the ability to form biofilms, thus excluding the influence of the serotype to which the strains belong.

## 5. Conclusions

Considering the results obtained in the present study, it can be concluded that chicken carcasses and cuts represent a major reservoir of *L. monocytogenes*. Furthermore, the samples analyzed suggest that chicken meat may have questionable hygienic and sanitary quality from the day of slaughter. The strains of *L. monocytogenes* isolated had a high degree of resistance to antibiotics and were capable of forming biofilms on polystyrene surfaces. Moreover, the correlation observed between the number of antibiotics to which the strains are resistant and their ability to form biofilms is worrying since it suggests that those strains capable of forming the most robust biofilms, and thus most easily able to contaminate foods, are also those with the greatest resistance to antibiotics and hence the most difficult to combat in case of infection. Nevertheless, it is also reassuring that the antibiotics most commonly used in clinical practice to treat infections caused by *L. monocytogenes* continue to be highly effective against the strains isolated.

## Figures and Tables

**Table 1:** Category, name, abbreviation and concentration of antibiotics used in evaluating resistance. The reference employed in classifying strains as susceptible, of reduced susceptibility or resistant is included.

Category		Antibiotic	Abbreviation	Concentration	Standard References
Beta-lactams	Penicillins	Ampicillin	AMP	10 µg	*Enterococcus* spp. [25]
		Oxacillin	OX	1 µg	*Staphylococcus* spp. [25]
	Cephalosporins	Cefoxitin	FOX	30 µg	*Staphylococcus* spp. [25]
		Cefotaxime	CTX	30 µg	*Streptococcus* spp. [26]
		Cefepime	FEP	30 µg	*Streptococcus* spp. [26]
Aminoglycosides		Gentamycin	CN	10 µg	*Staphylococcus* spp. [26]
Macrolides		Erythromycin	E	15 µg	*L. monocytogenes* [26]
Glycopeptides		Vancomycin	VA	30 µg	*Enterococcus* spp. [26]
Sulfonamides		Trimethoprim-sulfamethoxazole	SXT	25 µg	*L. monocytogenes* [26]
Rifamycins		Rifampicin	RD	5 µg	*Staphylococcus* spp. [26]
Tetracyclines		Tetracycline	TE	30 µg	*Staphylococcus* spp. [26]
Phenicols		Chloramphenicol	C	30 µg	*Staphylococcus* spp. [26]
Quinolones		Ciprofloxacin	CIP	5 µg	*Staphylococcus* spp. [26]
		Enrofloxacin	ENR	5 µg	*Staphylococcus* spp. [27]
Nitrofurans		Nitrofurantoin	F	300 µg	*Staphylococcus* spp. [25]

**Table 2:** Mean values and standard deviation (expressed as log_10_ cfu/g) for total aerobic counts and for psychrotrophic microorganisms of carcasses and cuts of each abattoir (A or B).

Abattoir	Total Aerobic Counts		Psychrotrophic Microorganisms	
	Carcasses	Cuts	Carcasses	Cuts
A	5.13 ± 0.33 ^a^_b_	5.86 ± 0.62 ^b^_a_	4.76 ± 0.48 ^a^_a_	4.86 ± 0.31 ^a^_a_
B	4.73 ± 0.24 ^a^_a_	5.83 ± 0.68 ^b^_a_	4.53 ± 0.67 ^a^_a_	5.56 ± 0.31 ^b^_b_
Both	4.93 ± 0.35	5.84 ± 0.64	4.65 ± 0.68	5.21 ± 0.33

Data in the same row with no superscript letters in common are significantly different (*p* < 0.05). Data in the same column with no subscript letters in common are significantly different (*p* < 0.05). The data for total aerobic counts and psychrotrophic microorganisms were considered separately.

**Table 3:** Resistance phenotypes were observed in 75 *Listeria monocytogenes* isolates from chicken tested against 15 clinically important antibiotics.

Number of Antibiotics	Number of Antibiotic Categories	Resistance Phenotype	Number of Isolates
4	2	OX-FOX-CTX-FEP	37
5	3	OX-FOX-CTX-FEP-RD	9
5	3	OX-FOX-CTX-FEP-CIP	1
5	3	OX-FOX-CTX-FEP-SXT	1
6	3	OX-FOX-CTX-FEP-CIP-ENR	1
6	4	OX-FOX-CTX-FEP-SXT-RD	1
8	6	OX-FOX-CTX-FEP-CN-E-RD-TE	16
9	7	OX-FOX-CTX-FEP-CN-E-SXT-RD-TE	8
10	8	OX-FOX-CTX-FEP-CN-E-SXT-RD-TE-CIP	1

OX (oxacillin; 1 µg), FOX (cefoxitin; 30 µg), CTX (cefotaxime; 30 µg), FEP (cefepime; 30 µg), CN (gentamycin; 10 µg), E (erythromycin; 15 µg), SXT (trimethoprim-sulfamethoxazole; 25 µg), RD (rifampicin; 5 µg), TE (tetracycline; 30 µg), CIP (ciprofloxacin; 5 µg), ENR (enrofloxacin; 5 µg).

## Data Availability

The data presented in this study are available on request from the corresponding author.

## Abbreviations

HACCP	Hazard Analysis Critical Control Points.
TAC	Total aerobic counts
PCA	Plate count agar
OCLA	Oxoid Chromogenic Listeria Agar
TSB	Tryptone Soy Broth
TSA	Tryptone Soy Agar
TE	tris-ethylenediamine-tetra-acetic acid
PCR	polymerase chain reaction
DNA	deoxyribonucleic acid
MHB	Mueller Hinon Broth
MHA	Mueller Hinton Agar
AMP	Ampicillin
OX	Oxacillin
FOX	Cefoxitin
CTX	Cefotaxime
FEP	Cefepime
CN	Gentamycin
E	Erythromycin
VA	Vancomycin
SXT	Trimethoprim-sulfamethoxazole
RD	Rifampicin
TE	Tetracycline
C	Chloramphenicol
CIP	Ciprofloxacin
ENR	Enrofloxacin
F	Nitrofurantoin
CLSM	Confocal Laser Scanning Microscopy
ANOVA	analysis of variance
MDR	multidrug-resistant
